# Knowledge, attitudes and practices of the Saudi population toward people with epilepsy: narrow or wide?

**DOI:** 10.1186/s42494-023-00140-5

**Published:** 2023-12-08

**Authors:** Alawi A. Al-Attas, Omar K. Alshehri, Abdulrahman G. Malhan, Hani M. Alabdaly, Osamah K. Alfentokh, Amen A. Bawazir

**Affiliations:** 1 Department of Adult Neurology, King Saud Medical City, 7790 Al Imam Abdul Aziz Ibn Muhammad Ibn Saud, Ulaishah, Riyadh 12746 3617, Riyadh, 12746 Saudi Arabia; 2 Department of Adult Neurology, National Neuroscience Institute, King Fahad Medical City, Riyadh, 11525 Saudi Arabia; 3 College of Medicine, ALMaarefa University, Riyadh, 13713 Saudi Arabia; 4 Department of Internal Medicine, King Abdullah Hospital, Bisha, 1108 Saudi Arabia; 5 Department of General Surgery, King Saud Medical City, Riyadh, 12746 Saudi Arabia

**Keywords:** Saudi population, Negative attitude, Knowledge, PWE, Public gap, Saudi Arabia

## Abstract

**Background:**

Although epilepsy has been acknowledged as an illness since ancient time, the public attitude toward epilepsy has not changed drastically due to the lack of appropriate information. This study aims to determine the public perceptions of epilepsy in five main regions of Saudi Arabia.

**Methods:**

In this cross-sectional questionnaire-based study, Saudi citizens living in the major cities of the five main regions of the Kingdom of Saudi Arabia (the central, eastern, western, northern, and southern regions) completed the survey that included 27 questions about epilepsy awareness.

**Results:**

A total of 7118 individuals from five regions participated in the survey: 6749 (94.8%) of respondents were Saudi, and 369 (5.2%) were non-Saudi. Of the 7118 respondents, 6897 respondents (96.9%) did not have epilepsy, and 3378 respondents (47.5%) stated that they had never witnessed an epileptic episode. In addition, 3749 (52.7%) respondents incorrectly attributed epilepsy to non-neurologic conditions, and 4865 (68.3%) respondents had an overall negative attitude toward people with epilepsy (PWE). They expressed objections to their child associating with PWE (2002; 28.1%) or their close relatives marrying PWE (3192; 44.8%), some believed that PWE are inferior (402; 5.6%), refused to hire a PWE (1126; 15.8%), and would feel discomfort around PWE (724; 10.3%). In addition, 2025 individuals (28.4%) believed that the health of PWE deteriorates over time; 1485 individuals (20.9%) believed that PWE get sick more often than others; 181 individuals (2.5%) believed that epilepsy could be transmitted; and 568 individuals (8.0%) believed that epilepsy could progress to malignant disease.

**Conclusions:**

In Saudi Arabia, there is a substantial need for increasing the awareness and improving education on epilepsy in order to reduce stigma and increase the understanding of epilepsy. Such efforts could help improve the lives of people afflicted with the disease and ultimately contribute to better health outcomes for the entire community.

## Background

Epilepsy is a neurological disorder characterized by recurrent seizures, affecting people of all ages [1]. Over 50 million individuals worldwide suffer from epilepsy, 80% of whom reside in low- and middle-income nations. The prevalence of epilepsy in 2017 was 8.75 per 1000 people in developing countries and 5.18 per 1000 people in developed countries [2, 3]. The median prevalence of active epilepsy in the Arabian Peninsula is 4.4 per 1000 people [4]. In the most recent survey on the prevalence of active epilepsy in the Kingdom of Saudi Arabia (KSA) conducted in 2001, the prevalence was approximately 6.5 cases per 1000 people [5].

Although epilepsy is one of the world’s oldest recognized disorders with written records dating back to 4000 B.C., the present public perception of epilepsy has not changed significantly. There is still fear, misunderstanding, prejudice, and social stigma toward epilepsy. Myths and false beliefs continue to dominate the public perception of the nature of epilepsy [6].

Numerous regional surveys have been conducted in Saudi Arabia to determine the public awareness of epilepsy patients. However, most previous studies utilized small sample sizes [7–11]. Most of these studies found that the education level and knowledge on epilepsy among Saudis living in cities need to be improved. Furthermore, earlier research did not analyze the elements that influence the knowledge gap or the attitudes toward patients with epilepsy [12].

In this study, we set out to assess the public knowledge gap, attitudes, and practices toward epilepsy patients in Saudi Arabia, with a particular emphasis on identifying potential areas for future intervention.

## Methods

### Study design and settings

A cross-sectional survey was conducted in the main cities of five main regions (central, eastern, western, southern, and northern) in Saudi Arabia in December 2019, lasting for four weeks. Due to the unfortunate delay caused by the detection of the first case of coronavirus disease 2019 in Saudi Arabia in March 2020, the statistical analysis was not concluded until September 2021.

In each of the five regions, a major urban center was used to represent the region. For example, Riyadh represented the central region, Jeddah the western, Dammam the eastern, Abha the southern, and Tabuk the northern. A sample size of 7500 individuals was considered adequate to adequately represent the entire nation. A total of 7118 responses were received, with a response rate of 94.9%. Saudis or non-Saudis aged  ≥ 18 years regardless of the gender, were recruited from major shopping centers, universities, and workplaces. Those who were too ill to provide the requested information were excluded from the study. Before completing the questionnaire, all participants provided informed consent to be enrolled in the study.

### Study variables and operational definitions

The standard, validated, self-administered questionnaire pertained to epilepsy awareness and knowledge as well as attitudes and practices toward those with epilepsy. The questionnaire was adopted from previous studies to obtain the necessary data [7], and consisted of the following questions: sociodemographics of the participants (8 questions); knowledge of epilepsy and attitude toward people with epilepsy (PWE) (11 questions); abilities of persons with epilepsy (11 questions); and five questions related to concepts of health and disease related to PWE. The average number of correct or positive responses was considered. The document was translated into Arabic. The English version was also kept for English-speaking participants. The questionnaire was distributed through 35 medical student volunteers living in different cities in the major regions of Saudi Arabia. The study was supervised by three senior investigators: a professor of epidemiology and public health and two consultants at the Department of Adult Neurology at King Saud Medical City. Data were acquired through face-to-face interactions. The data collectors were instructed to know the significance of gathering responses from a representative sample of the public to accurately capture the knowledge of the broader population. 

A pilot study was conducted with 40 nonparticipants in the main study to verify the internal consistency and reliability of Arabic and English versions. The two versions were readily available for distribution to all Arabic and English speakers. The pilot participants were requested to provide input based on their cultures and values. Based on their feedback, some irrelevant questions were eliminated, and others were adjusted to fit the local context. Regarding the scale reliability, the Cronbach’s alpha value for attitude was 0.73 and that for knowledge was 0.81.

The first section of the sociodemographic characteristics of the participants included nationality, age, gender, level of education, type of living area, region, occupation, and monthly income. The other sections consisted of questions with yes-or-no answers.

### Statistical analysis

The data analysis was conducted using the SPSS (version 23.0, IBM SPSS Inc., Armonk, NY) software. Demographic information and epilepsy knowledge and attitude scores (mean, percentage, and frequency distribution) were evaluated. The relationships between demographic data and knowledge/attitude scores were analyzed using chi-square test. For those with a *P*-value < 0.05, a regression analysis was conducted and adjusted using a backward logistic regression test. In this study, a *P*-value of < 0.05 was considered as statistically significant.

This study was approved by the Institutional Review Board (IRB) and Ethics Committee (IRB Log number: 18-419E). Participation was voluntary, and replies were kept confidential.

## Results

### Demographics of participants

Table 1 illustrates the sociodemographic characteristics of the 7118 participants in this study. Most of the participants were Saudi (94.8%), young (< 30 years) (61.0%), female (65.3%), university graduates (74.7%), and residents of major cities (70.4%), A proportion of participants were from the Eastern Region (32.0%) and students (46.7%), and a high income (38.1%).

**Table 1:** Sociodemographic characteristics of the participants (*n* = 7118)

		*n*	Proportion (%)
Nationality	Saudi	6749	94.8
	Non-Saudi	369	5.2
Age (years)	18–29	4345	61.0
	≥ 30	2773	39.0
Gender	Male	2469	34.7
	Female	4649	65.3
Education	Secondary/diploma	1802	25.3
	University and above	5316	74.7
Residential area	Main city	5009	70.4
	Minor city	1064	14.9
	Village	1045	14.7
Region	Central Region	1177	16.5
	Eastern Region	2281	32.0
	Western region	1603	22.5
	Northern Region	402	5.6
	Southern Region	1655	23.3
Occupation	Unemployed	1415	19.9
	Student	3323	46.7
	Retired	426	6.0
	Employee	1954	27.5
Income per month (SR)	< 4000	2044	28.7
	4000–10,000	2363	33.2
	> 10,000	2711	38.1

### Knowledge and attitude toward epilepsy among the participants

Among the surveyed participants, approximately 221 reported that they had a history of epilepsy (3.1%), and 159 of them were under treatment (2.2%) for weak memory. Almost all surveyed people had heard or read about epilepsy (94.4%). Additionally, approximately half (52.5%) had witnessed an epileptic seizure or knew someone with epilepsy (55.4%). Only 10.9% of participants provided incorrect answers about the nature of an epileptic attack, picking the option that epilepsy was attributed to a non-neurological disease (Table 2). Moreover, approximately 65.8% of the participants showed less knowledge of epilepsy.

**Table 2:** General knowledge and attitude toward people with epilepsy

Variables	Negative answers	%
Are you a person with epilepsy?	6897	96.9
Do you have weak memory or take drugs for that?	6959	97.8
Have you ever heard or read about epilepsy?	397	5.6
Have you ever seen anyone having an epileptic seizure?	3378	47.5
Have you ever known anyone who had epilepsy?	3173	44.6
What do you think an epileptic attack is?	3749	52.7
**Overall less knowledge**	**4686**	**65.8**
**Positive attitude**		
Objection to a person with epilepsy associating with your child.	2002	28.1
Objection to a person with epilepsy marrying your close relative.	3192	44.8
Do you think a person with epilepsy is an inferior person?	402	5.6
If you have a company, would you hire a person with epilepsy?	5992	84.2
Would you feel uncomfortable with a person with epilepsy?	724	10.2
**Overall positive attitude**	**4465**	**62.7**

Although our study generally revealed a predominantly positive attitude among 62.7% of participants, more than a quarter of the participants (28.1%) expressed opposition to their children associating with a person with a history of epileptic seizures, and almost half of the participants (44.8%) expressed opposition to PWE marrying close relatives. As shown in Table 2, the majority of participants did not regard epileptic people as inferior to other people of the community (94.4%), and most expressed willingness to hire an epileptic person (84.2%) or would not feel uncomfortable with an epileptic person (89.8%).

The overall negative abilities of PWE reported by the participants was 43.6%. As shown in Table 3, with regard to the abilities and functionality of PWE, the majority of the participants considered that PWE could have children (97.2%), were not of low intelligence (95.8%), could participate in similar social activities as others (90.1%), could occupy the same jobs as other people (77.5%), and feel full of energy and have high spirits (69.3%). In addition, 32.2% of the participants thought that PWE are usually emotionally disturbed, which affect their social life, and 34.3% of the participants thought that the emotional problems interfere with their normal social activities. However, some participants answered that PWE would spend a greater amount of time on work than others (43.6%), so they accomplish fewer tasks than others (62.2%). About half of the participants thought that a person with epilepsy could not drive a car (43.5%).

**Table 3:** The negative answers of participants about the abilities of people with epilepsy

Variables	Negative answers	%
Do you think people with epilepsy can have children?	199	2.8
Do you think people with epilepsy are of low intelligence?	296	4.2
Does a person with epilepsy have social activities like others?	706	9.9
The capability of people with epilepsy in employment is the same as others.	1601	22.5
Do people with epilepsy feel full of energy and high spirits?	2188	30.7
Do people with epilepsy have emotional disturbances that affect their social life?	2292	32.2
The emotional problems of people with epilepsy interfere with normal social activities.	2443	34.3
People with epilepsy spend the same amount of time on work as others.	3104	43.6
Do people with epilepsy accomplish less than others?	4424	62.2
Can a person with epilepsy drive?	3097	43.5
**Overall negative abilities of persons with epilepsy**	**3105**	**43.6**

Regarding health- and disease-related concepts, as shown in Table 4, more than half of the participants believed that a person with epilepsy is as healthy as anyone else (57.2%), does not deteriorate over time (71.6%), and does not get sick more easily than others (79.1%). Most participants believed that epilepsy is not a contagious disease (97.5%). On the other hand, a minority of participants thought the disease could be malignant (8%).

**Table 4:** Health and disease-related concepts for people with epilepsy

Is a person with epilepsy as healthy as anyone else?	No	3044	42.8
Does the health of people with epilepsy get worse over time?	Yes	2025	28.4
Do people with epilepsy get sick easier than other people?	Yes	1485	20.9
Is epilepsy contagious?	Yes	181	2.5
Does epilepsy develop into malignant disease?	Yes	568	8.0

The overall knowledge of the participants was scored from six questions related to the knowledge of epilepsy and PWE: “score 1, good knowledge” and “score 0, poor knowledge”. This bivariate scale was used to compute the unadjusted and adjusted odd ratios in relation to the sociodemographic characteristics of the participants, as shown in Table 5. Participants with non-Saudi nationality, younger age (< 30 years old), male gender, or educational level of secondary school or below, as well as those from an eastern, northern, or western region, or those categorized as students were more likely to have poor knowledge of epilepsy and the nature of the disease than their counterparts. Other factors related to the type of residence or level of income per month did not influence the participants’ level of knowledge.

**Table 5:** Regression analysis of the overall negative knowledge among participants by different sociodemographic characteristics

		OR	95% CI	*P* value	AOR	95% CI	*P *value
Nationality	Saudi	Reference	-	-	Reference	-	-
	Non-Saudi	1.323	1.014–1.725	0.039			
Age (years)	18–29	1.276	1.089–1.496	0.003	1.293	1.104–1.514	< 0.001
	≥ 30	Reference	-	-	-	-	-
Gender	Male	1.163	1.027–1.318	0.018	1.161	1.026–1.314	0.018
	Female	Reference	-	-	Reference	-	**-**
Education	Secondary/Diploma	1.296	1.131–1.484	< 0.001	1.312	1.148–1.499	< 0.001
	University & above	Reference	-	-	Reference	-	-
Residential area	Main city	1.091	0.922–1.290	0.310	-	-	-
	Minor city	0.939	0.764–1.155	0.554	-	-	-
	Village	Reference	-	-	-	-	-
Region	Central	Reference	-	-	Reference	-	-
	Eastern	1.214	1.011–1.456	0.037	1.041	0.887–1.221	0.624
	Western	1.178	1.010–1.374	0.037	0.947	0.797–1.125	0.535
	Northern	1.279	1.075–1.522	0.005	1.232	0.940–1.615	0.131
	Southern	1.004	0.767–1.315	0.977	1.252	1.052–1.492	0.012
Occupation	Unemployed	Reference	-	-	Reference	-	-
	Student	0.756	0.634–0.900	0.002	1.096	0.930–1.291	0.275
	Retired	1.135	0.814–1.582	0.455	1.430	1.202–1.701	< 0.001
	Employee	1.061	0.897–1.256	0.490	0.856	0.682–1.074	0.178
Income per month	Below 4000 SR	0.959	0.829–1.110	0.578	-	-	-
	From 4000 to 10,000 SR	0.955	0.839–1.088	0.488	-	-	-
	More than 10,000 SR	Reference	-	-	-	-	-

*OR* Odd ratio, *95% CI* 95% confidence interval, *AOR* Adjusted odds ratio

A similar approach was used to analyze acceptable and unacceptable attitudes toward PWE. The scales of five questions were computed and used for further analysis to calculation of the unadjusted and adjusted odds ratios. Table 6 illustrates the association between the demographic characteristics and the negative attitude of the participants toward PWE. The unadjusted and unadjusted regression tests showed that participants who were non-Saudi, male with secondary schooling or below, or had retired people were more likely to have a negative attitude toward PWE. The other variables such as age, type of residence (main cities or others), region, and economic status, showed no influence on the attitudes toward PWE.

**Table 6:** Regression analysis of the overall negative attitude of participants by different sociodemographic characteristics and overall negative attitude

		OR	95% CI	*P* value	AOR	95% CI	*P* value
Nationality	Saudi	Reference	-	-	Reference	-	-
	Non-Saudi	1.284	1.004–1.642	0.046	1.305	1.024–1.664	0.032
Age (years)	18–29	0.997	0.858–1.159	0.972	-	-	-
	≥ 30	Reference	-	-	-	-	-
Gender	Male	1.288	1.146–1.449	< 0.001	1.287	1.145–1.446	< 0.001
	Female	Reference	-	-	Reference	-	-
Education	Secondary/Diploma	1.184	1.043–1.344	0.009	1.198	1.058–1.356	0.004
	University & above	Reference	-	-	Reference	-	-
Residential area	Main city	0.995	0.852–1.162	0.949	-	-	-
	Minor city	1.011	0.837–1.222	0.906	-	-	-
	Village	Reference	-	-	-	-	-
Region	Central	Reference	-	-	Reference	-	-
	Eastern	0.938	0.806–1.093	0.413	0.942	0.810–1.095	0.435
	Western	0.989	0.840–1.165	0.896	0.992	0.842–1.169	0.924
	Northern	1.262	0.979–1.627	0.073	1.275	0.989–1.643	0.060
	Southern	1.133	0.955–1.344	0.152	1.141	0.969–1.345	0.114
Occupation	Unemployed	Reference	-	-	Reference	-	-
	Student	0.988	0.842–1.160	0.886	1.007	0.863–1.175	0.001
	Retired	1.213	1.028–1.431	0.023	1.231	1.087–1.395	
	Employee	0.910	0.725–1.142	0.417	0.896	0.717–1.121	0.337
Income per month	Below 4000 SR	1.087	0.950–1.244	0.225	-	-	-
	From 4000 to 10,000 SR	1.064	0.942–1.201	0.319	-	-	-
	More than 10,000 SR	Reference	-	-	-	-	-

*OR* Odds ratio, *95% CI* 95% confidence interval, *AOR* Adjusted odds ratio

## Discussion

This is the first comprehensive, large-scale survey (7118 participants) to assess the knowledge, attitudes, and perceptions of the Saudi community toward PWE across all five main regions of Saudi Arabia.

The participants in this survey demonstrated low epilepsy knowledge (65.8%). However, this rate is higher than those reported in other research conducted in Saudi Arabia or elsewhere [13, 14]. The reason for this disparity is not clear; nevertheless, a clear knowledge of the meaning of epilepsy was obscured in our study because 47.5% of our participants had never seen a person with epilepsy.

Several studies have been conducted to assess the awareness of epilepsy in Saudi Arabia and other Arab countries. They revealed some interesting similarities and differences in the level of knowledge and attitudes toward epilepsy across the region. Furthermore, studies on the knowledge and attitudes toward epilepsy have also been conducted in developed and Western countries as well as the Arab world [7–12]. In Saudi Arabia, for instance, a study involving 1044 participants found that the vast majority of participants had heard of epilepsy, although 60.7% of them demonstrated ignorance of the condition and an inappropriate attitude when interacting with someone having a seizure [11]. Our study showed a generally weak level (65.8%) of knowledge and a negative attitude (68.3%) among the participants about epilepsy. However, this low level of knowledge in our study is consistent with other studies in the United States and Ethiopia, which reported a low level of knowledge on epilepsy [15].

This divergence between our study and others may be attributed to the predominance of young participants in this study who lacked prior expertise with epilepsy patients, as revealed by the adjusted regression analysis (AOR = 1.293, 95% CI = 1.104–1.514). Similarly, this study indicated a higher proportion of negative attitudes than earlier studies (68.3%). Non-Saudis, for instance, were found to be more likely to hold unfavorable opinions than Saudis (AOR = 1.305; 95% CI = 1.024–1.664), males had more negative attitudes than females (AOR = 1.287; 95% CI = 1.145–1.446), and lower educated individuals with lower education had more negative attitudes than university graduates (AOR = 1.198; 95% CI = 1.058–1.346).

Similar to our findings, many studies conducted in Arab nations regarding the knowledge and attitudes about PWE have demonstrated that the gap has not changed significantly [16, 17]. Two studies in Jordan identified the presence of negative attitudes toward epilepsy, similar to our findings. A study conducted in the United Arab Emirates (UAE) in 1998 revealed that the majority of respondents were familiar with epilepsy. In addition, educational level and age were shown to be positively correlated with the awareness of the condition. Some people believe that there is no cure, while others support cauterization and faith healing as treatment options [18]. Moreover, in a 2016 survey conducted in the UAE, most of the participants had heard of epilepsy, and 80% had a positive attitude toward social acceptance. Despite this, 13.9% of respondents believed that PWE should be isolated [19]. In a study conducted in Egypt, all participants were familiar with epilepsy. A specific comparison revealed that persons with a family member with epilepsy had more positive attitudes. In addition, Egyptian pupils possessed higher levels of social acceptance and positive attitudes [20]. Similarly, recent research conducted in Tunisia revealed that epilepsy-related knowledge and attitudes of the public were satisfactory. However, there are still negative attitudes and misunderstandings [21].

A study conducted in Kuwait in 2008 found that most people were aware of epilepsy and that roughly half of them had interacted with someone who had epilepsy. A larger proportion expressed objection to marrying PWE, while a smaller proportion believed that putting something in a patient’s mouth to prevent tongue biting during a seizure was inappropriated [14]. Although our study generally reveals a positive attitude at approximately 62.7%, we also found two primary objections for a person with epilepsy getting associated with one’s child (28.1%) and a person with epilepsy marrying one close relative (44.8%). The remaining three questions indicate a significantly lower level of objection toward individuals with epilepsy. It is worth noting that this attitude is relatively positive when compared to many other communities worldwide. There are still misconceptions about epilepsy, even though knowledge, attitudes, and beliefs about the condition have improved significantly. False beliefs about epilepsy have resulted in stigmatizing attitudes toward PWE in numerous communities throughout the history [22]. A recent study conducted in Palestine examined the knowledge and attitudes of physical educators regarding students with epilepsy and the disorder. The results showed that physical educators in Palestine had inadequate knowledge and negative attitudes toward epilepsy and students with epilepsy [23]. Al-Khateeb et al. [24] examined the knowledge and attitudes of more than 46,000 people from diverse backgrounds (the public, physicians, nurses, school instructors and counsellors, students, and PWE) in 16 studies conducted in Kuwait, Jordan, Egypt, Saudi Arabia, Oman, and the UAE. They found widespread misconceptions about epilepsy, such as the belief that "epilepsy is untreatable" [7] and that "it is a psychiatric disorder" [14, 25, 26]. Seven studies reported inadequate knowledge of epilepsy [7, 14, 18, 27, 28], while four studies reported average knowledge [19, 20, 23, 29]. Two studies reported moderately positive attitudes [18, 19].

Overall, these studies provide valuable insights into the public awareness about epilepsy in Saudi Arabia and other Arab countries and highlight the significant need for increased awareness and education about epilepsy across the Arab world. While there may be some regional differences in the awareness and attitudes toward epilepsy, it is necessary to increase education and awareness campaigns to address the knowledge gaps and negative attitudes that persist in many communities.

There are some merits and limitations in this study. The primary strengths are the size and the representativeness of the samples surveyed. Our findings can therefore be extrapolated to the entire Saudi population. This study may provide reference for educational initiatives proposed by the Ministry of Health in Saudi Arabia, which would bolster epilepsy education within the general population. This would ultimately advance comprehension of epilepsy and, consequently, enhance the quality of life of individuals living with epilepsy. The present study did not specifically investigate variances in response between participants from urban and rural areas in certain aspects because this was not a primary variable of interest in this study. Instead, this study recognizes the importance of this distinction and recommend its inclusion in future studies to better discern concepts originating from different contexts. There are some limitations of this study, including the open-ended nature of the questionnaire which may have led to imprecise answers. In addition, the cross-sectional study design precludes the establishment of a causal connection between research variables.

## Conclusions

Based on existing research and data, it appears that there is a substantial lack of understanding of epilepsy and unfavorable attitudes toward PWE in Saudi Arabia. It is crucial to raise the awareness about epilepsy in Saudi Arabia. Public health programs should focus on public education on the symptoms, complications, management, and societal effects of epilepsy as well as how to combat the stigma linked to epilepsy.

Saudi Arabia should consider implementing initiatives such as media campaigns and school-based health education to boost epilepsy awareness. Future researches including longitudinal studies, demographic and regional analyses, tailored interventions, and rigorous evaluations of educational programs are needed to assess the impact and effectiveness of these initiatives, identify challenges, and explore the use of digital platforms for improving public education.

## Data Availability

The corresponding author takes full responsibility for the data, has full access to all data, and has the right to publish all data separate and apart from any sponsor.

## Abbreviations

PWE: People with epilepsy
KSA: Kingdom of Saudi Arabia
UAE: United Arab Emirates
